# 3-Cyano­anilinium chloride

**DOI:** 10.1107/S1600536808020485

**Published:** 2008-07-12

**Authors:** Xiao-Chun Wen

**Affiliations:** a Ordered Matter Science Research Center, College of Chemistry and Chemical Engineering, Southeast University, Nanjing 210096, People’s Republic of China

## Abstract

In the title salt, C_7_H_7_N_2_^+^·Cl^−^, all non-H atoms of the cation are essentially coplanar (r.m.s. deviation = 0.005 Å). In the crystal structure, the organic cations and chloride ions are linked to form a two-dimensional network parallel to the (001) plane by N—H⋯Cl hydrogen bonds.

## Related literature

For the use of amine derivatives in coordination chemistry, see: Manzur *et al.* (2007 ▶); Ismayilov *et al.* (2007 ▶); Austria *et al.* (2007 ▶).
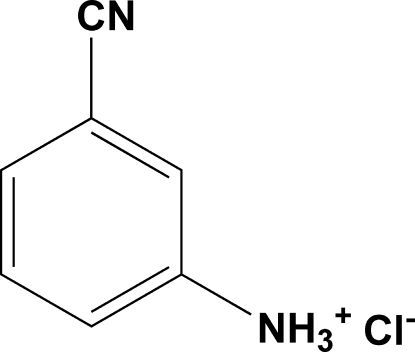


## Experimental

### 

#### Crystal data

C_7_H_7_N_2_^+^·Cl^−^
*M* *_r_* = 154.60
Triclinic, 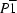

*a* = 4.663 (3) Å
*b* = 6.074 (5) Å
*c* = 13.212 (9) Å
α = 93.37 (5)°
β = 96.201 (19)°
γ = 96.22 (4)°
*V* = 368.9 (5) Å^3^
*Z* = 2
Mo *K*α radiation
μ = 0.43 mm^−1^
*T* = 298 (2) K
0.25 × 0.18 × 0.18 mm

#### Data collection

Rigaku Mercury2 diffractometer
Absorption correction: multi-scan (*CrystalClear*; Rigaku, 2005 ▶) *T*_min_ = 0.901, *T*_max_ = 0.917
2486 measured reflections
1618 independent reflections
1342 reflections with *I* > 2σ(*I*)
*R*_int_ = 0.021

#### Refinement

*R*[*F* ^2^ > 2σ(*F* ^2^)] = 0.052
*wR*(*F* ^2^) = 0.157
*S* = 1.07
1618 reflections
91 parameters
H-atom parameters constrained
Δρ_max_ = 0.52 e Å^−3^
Δρ_min_ = −0.52 e Å^−3^

### 

Data collection: *CrystalClear* (Rigaku, 2005 ▶); cell refinement: *CrystalClear*; data reduction: *CrystalClear*; program(s) used to solve structure: *SHELXS97* (Sheldrick, 2008 ▶); program(s) used to refine structure: *SHELXL97* (Sheldrick, 2008 ▶); molecular graphics: *SHELXTL* (Sheldrick, 2008 ▶); software used to prepare material for publication: *SHELXTL*.

## Supplementary Material

Crystal structure: contains datablocks I, global. DOI: 10.1107/S1600536808020485/ci2624sup1.cif

Structure factors: contains datablocks I. DOI: 10.1107/S1600536808020485/ci2624Isup2.hkl

Additional supplementary materials:  crystallographic information; 3D view; checkCIF report

## Figures and Tables

**Table 1:** Hydrogen-bond geometry (Å, °)

*D*—H⋯*A*	*D*—H	H⋯*A*	*D*⋯*A*	*D*—H⋯*A*
N2—H9*A*⋯Cl1^i^	0.89	2.61	3.111 (3)	116
N2—H9*A*⋯Cl1^ii^	0.89	2.65	3.178 (3)	119
N2—H9*C*⋯Cl1^iii^	0.89	2.73	3.278 (3)	121
N2—H9*B*⋯Cl1	0.89	2.76	3.338 (3)	124

Symmetry codes: (i) 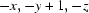; (ii) 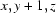; (iii) 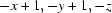.

**Fig. 1.**
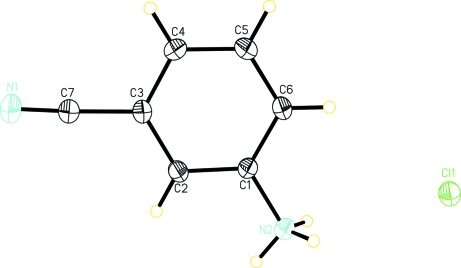
A view of the title compound with the atomic numbering scheme. Displacement ellipsoids are drawn at the 30% probability level.

**Fig. 2.**
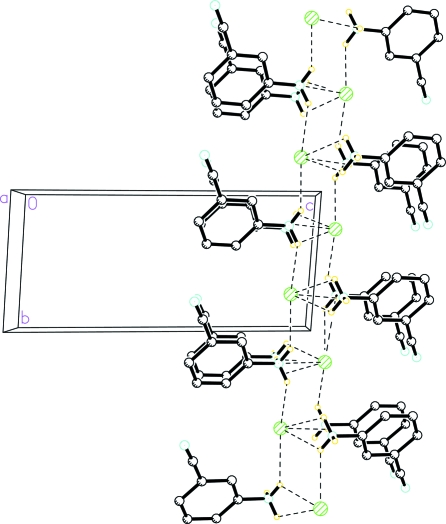
Part of the crystal packing of the title compound viewed along the a axis. H atoms not involved in hydrogen bonding (dashed lines) have been omitted for clarity.

## supplementary crystallographic information

### Comment

In the past five years, we have focused on the chemistry of amine derivatives because of their multiple coordination modes as ligands to metal ions and for the construction of novel metal-organic frameworks (Manzur *et al.* 2007; Ismayilov *et al.* 2007; Austria *et al.* 2007). We report here the crystal structure of the title compound, 3-cyanobenzenaminium monochloride.

In the title compound (Fig.1), the N2 atom of the amine group is protonated. The nitrile group is coplanar with the benzene ring. Bond lengths and angles lie within normal ranges.

In the crystal structure the organic cation and Cl^-^ ions are linked to form a two-dimensional network parallel to the (0 0 1) plane (Fig.2) by N—H···Cl hydrogen bonds (Table 1).

### Experimental

3-Cyanobenzenaminium monochloride (3 mmol) was dissolved in ethanol (20 ml). The solution was allowed to evaporate to obtain colourless block-shaped crystals of the title compound.

### Refinement

All H atoms were fixed geometrically [C-H = 0.93 Å and N-H = 0.89 Å] and treated as riding, with *U*_iso_(H) = 1.2U_eq_(C) and 1.5U_eq_(N).

### Crystal data

**Table:**

C_7_H_7_N_2_^+^·Cl^–^	*Z* = 2
*M**_r_* = 154.60	*F*_000_ = 160
Triclinic, *P*1	*D*_x_ = 1.392 Mg m^−^^3^
Hall symbol: -P 1	Mo *K*α radiation λ = 0.71073 Å
*a* = 4.663 (3) Å	Cell parameters from 1678 reflections
*b* = 6.074 (5) Å	θ = 2.3–24.4º
*c* = 13.212 (9) Å	µ = 0.44 mm^−^^1^
α = 93.37 (5)º	*T* = 298 (2) K
β = 96.201 (19)º	Block, colourless
γ = 96.22 (4)º	0.25 × 0.18 × 0.18 mm
*V* = 368.9 (5) Å^3^	

### Data collection

**Table:**

Rigaku Mercury2 diffractometer	1618 independent reflections
Radiation source: fine-focus sealed tube	1342 reflections with *I* > 2σ(*I*)
Monochromator: graphite	*R*_int_ = 0.021
Detector resolution: 13.6612 pixels mm^-1^	θ_max_ = 27.5º
*T* = 298(2) K	θ_min_ = 3.1º
ω scans	*h* = −6→6
Absorption correction: multi-scan(CrystalClear; Rigaku, 2005)	*k* = −7→5
*T*_min_ = 0.901, *T*_max_ = 0.917	*l* = −16→17
2486 measured reflections	

### Refinement

**Table:**

Refinement on *F*^2^	Secondary atom site location: difference Fourier map
Least-squares matrix: full	Hydrogen site location: inferred from neighbouring sites
*R*[*F*^2^ > 2σ(*F*^2^)] = 0.052	H-atom parameters constrained
*wR*(*F*^2^) = 0.157	*w* = 1/[σ^2^(*F*_o_^2^) + (0.0787*P*)^2^ + 0.2472*P*] where *P* = (*F*_o_^2^ + 2*F*_c_^2^)/3
*S* = 1.07	(Δ/σ)_max_ = 0.001
1618 reflections	Δρ_max_ = 0.52 e Å^−^^3^
91 parameters	Δρ_min_ = −0.52 e Å^−^^3^
Primary atom site location: structure-invariant direct methods	Extinction correction: none

### Special details

**Table:**

Geometry. All e.s.d.'s (except the e.s.d. in the dihedral angle between two l.s. planes) are estimated using the full covariance matrix. The cell e.s.d.'s are taken into account individually in the estimation of e.s.d.'s in distances, angles and torsion angles; correlations between e.s.d.'s in cell parameters are only used when they are defined by crystal symmetry. An approximate (isotropic) treatment of cell e.s.d.'s is used for estimating e.s.d.'s involving l.s. planes.
Refinement. Refinement of *F*^2^ against ALL reflections. The weighted *R*-factor *wR* and goodness of fit *S* are based on *F*^2^, conventional *R*-factors *R* are based on *F*, with *F* set to zero for negative *F*^2^. The threshold expression of *F*^2^ > σ(*F*^2^) is used only for calculating *R*-factors(gt) *etc*. and is not relevant to the choice of reflections for refinement. *R*-factors based on *F*^2^ are statistically about twice as large as those based on *F*, and *R*- factors based on ALL data will be even larger.

### Fractional atomic coordinates and isotropic or equivalent isotropic displacement parameters (Å^2^)

**Table:**

	*x*	*y*	*z*	*U*_iso_*/*U*_eq_	
Cl1	0.38158 (16)	0.25924 (11)	0.07001 (5)	0.0414 (3)	
C1	0.0572 (5)	0.7649 (4)	0.19904 (18)	0.0302 (5)	
N2	0.1472 (5)	0.7573 (4)	0.09653 (16)	0.0377 (6)	
H9A	0.0747	0.8643	0.0622	0.057*	
H9B	0.0816	0.6259	0.0641	0.057*	
H9C	0.3402	0.7770	0.1008	0.057*	
C3	−0.1968 (5)	0.9320 (4)	0.3237 (2)	0.0329 (6)	
C7	−0.3747 (6)	1.0962 (5)	0.3564 (2)	0.0391 (6)	
C6	0.1466 (6)	0.6149 (5)	0.2669 (2)	0.0374 (6)	
H6A	0.2625	0.5081	0.2475	0.045*	
N1	−0.5144 (6)	1.2233 (5)	0.3856 (2)	0.0545 (7)	
C4	−0.1099 (6)	0.7815 (5)	0.3928 (2)	0.0395 (6)	
H4A	−0.1675	0.7865	0.4580	0.047*	
C2	−0.1148 (5)	0.9263 (4)	0.2256 (2)	0.0322 (6)	
H2A	−0.1732	1.0271	0.1794	0.039*	
C5	0.0624 (7)	0.6251 (5)	0.3633 (2)	0.0443 (7)	
H5A	0.1229	0.5248	0.4094	0.053*	

### Atomic displacement parameters (Å^2^)

**Table:**

	*U*^11^	*U*^22^	*U*^33^	*U*^12^	*U*^13^	*U*^23^
Cl1	0.0519 (5)	0.0373 (4)	0.0395 (4)	0.0155 (3)	0.0126 (3)	0.0062 (3)
C1	0.0334 (12)	0.0304 (13)	0.0285 (12)	0.0073 (10)	0.0074 (10)	0.0027 (9)
N2	0.0446 (13)	0.0398 (13)	0.0324 (12)	0.0139 (10)	0.0107 (10)	0.0040 (9)
C3	0.0308 (12)	0.0338 (14)	0.0360 (13)	0.0102 (10)	0.0063 (10)	0.0020 (10)
C7	0.0404 (14)	0.0421 (16)	0.0380 (14)	0.0140 (12)	0.0093 (12)	0.0045 (11)
C6	0.0442 (15)	0.0333 (14)	0.0388 (14)	0.0165 (11)	0.0094 (11)	0.0065 (11)
N1	0.0597 (17)	0.0551 (17)	0.0558 (17)	0.0274 (14)	0.0191 (14)	0.0036 (13)
C4	0.0491 (16)	0.0429 (16)	0.0307 (13)	0.0136 (12)	0.0130 (12)	0.0063 (11)
C2	0.0325 (13)	0.0316 (13)	0.0346 (13)	0.0099 (10)	0.0048 (10)	0.0062 (10)
C5	0.0577 (18)	0.0420 (17)	0.0398 (15)	0.0216 (13)	0.0132 (13)	0.0154 (12)

### Geometric parameters (Å, °)

**Table:**

C1—C6	1.380 (4)	C3—C7	1.440 (4)
C1—C2	1.385 (3)	C7—N1	1.139 (4)
C1—N2	1.460 (3)	C6—C5	1.374 (4)
N2—H9A	0.89	C6—H6A	0.93
N2—H9B	0.89	C4—C5	1.374 (4)
N2—H9C	0.89	C4—H4A	0.93
C3—C4	1.390 (4)	C2—H2A	0.93
C3—C2	1.391 (4)	C5—H5A	0.93
			
C6—C1—C2	121.9 (2)	C5—C6—C1	119.2 (2)
C6—C1—N2	119.8 (2)	C5—C6—H6A	120.4
C2—C1—N2	118.3 (2)	C1—C6—H6A	120.4
C1—N2—H9A	109.5	C5—C4—C3	119.1 (2)
C1—N2—H9B	109.5	C5—C4—H4A	120.4
H9A—N2—H9B	109.5	C3—C4—H4A	120.4
C1—N2—H9C	109.5	C1—C2—C3	117.5 (2)
H9A—N2—H9C	109.5	C1—C2—H2A	121.2
H9B—N2—H9C	109.5	C3—C2—H2A	121.2
C4—C3—C2	121.3 (2)	C6—C5—C4	121.0 (2)
C4—C3—C7	118.2 (2)	C6—C5—H5A	119.5
C2—C3—C7	120.4 (2)	C4—C5—H5A	119.5
N1—C7—C3	177.6 (3)		
			
C2—C1—C6—C5	0.2 (4)	N2—C1—C2—C3	−179.6 (2)
N2—C1—C6—C5	179.4 (3)	C4—C3—C2—C1	0.0 (4)
C2—C3—C4—C5	0.5 (4)	C7—C3—C2—C1	179.8 (2)
C7—C3—C4—C5	−179.3 (3)	C1—C6—C5—C4	0.3 (5)
C6—C1—C2—C3	−0.3 (4)	C3—C4—C5—C6	−0.6 (5)

### Hydrogen-bond geometry (Å, °)

**Table:**

*D*—H···*A*	*D*—H	H···*A*	*D*···*A*	*D*—H···*A*
N2—H9A···Cl1^i^	0.89	2.61	3.111 (3)	116
N2—H9A···Cl1^ii^	0.89	2.65	3.178 (3)	119
N2—H9C···Cl1^iii^	0.89	2.73	3.278 (3)	121
N2—H9B···Cl1	0.89	2.76	3.338 (3)	124

Symmetry codes: (i) −*x*, −*y*+1, −*z*; (ii) *x*, *y*+1, *z*; (iii) −*x*+1, −*y*+1, −*z*.
